# Effect of Dietary Nori (Dried Laver) on Blood Pressure in Young Japanese Children: An Intervention Study

**DOI:** 10.2188/jea.JE20190176

**Published:** 2021-01-05

**Authors:** Keiko Wada, Michiko Tsuji, Kozue Nakamura, Shino Oba, Sakiko Nishizawa, Keiko Yamamoto, Kaori Watanabe, Kyoko Ando, Chisato Nagata

**Affiliations:** 1 Department of Epidemiology and Preventive Medicine, Gifu University Graduate School of Medicine, Gifu, Japan; 2 Department of Food Science and Nutrition, Nagoya Women’s University, Nagoya, Japan; 3 Gifu City Healthcare Center, Gifu, Japan; 4 Graduate School of Health Sciences, Gunma University, Maebashi, Japan; 5 Department of Food and Culture Science, Aichi Bunkyo Women’s College, Aichi, Japan

**Keywords:** blood pressure, child, laver, nutrition, seaweed

## Abstract

**Background:**

Few studies have examined the association between seaweed intake and blood pressure in children. We conducted an intervention study to investigate whether seaweed intake affects blood pressure.

**Methods:**

Subjects were children aged 4 to 5 years attending a preschool in Aichi Prefecture, Japan, in 2010. Among 99 students, 89 (89.9%) were enrolled in our study. Nori (dried laver), an edible seaweed widely consumed in Japan, was used as a dietary intervention. Children in the intervention group were asked to consume 1.76 grams per day of roasted nori in addition to standard meals for 10 weeks. Children in the control group consumed their usual diet. Before the intervention and at the 10th week of the intervention, children’s blood pressure was measured three times successively using an automated sphygmomanometer with subjects in a sitting position. Changes in systolic (SBP) and diastolic blood pressure (DBP) were compared between 55 children in the intervention group and 26 in the control group after adjustment for SBP and DBP before the intervention.

**Results:**

Changes in SBP were −8.29 mm Hg in the intervention group and +0.50 mm Hg in the control group (*P* for difference in change = 0.051). Changes in DBP were −6.77 mm Hg in the intervention group and −0.05 mm Hg in the control group (*P* = 0.031). In girls, no difference in blood pressure changes was found between the intervention and control groups.

**Conclusion:**

Nori intake lowered DBP level in boys. Seaweed intake might have preventive effects on elevated blood pressure in childhood.

## INTRODUCTION

Several studies have shown that blood pressure is linked over time from childhood to adulthood and that atherogenesis begins in childhood.^1^^–^^3^ Increased blood pressure is an established risk factor of cardiovascular disease, which is a major cause of death among Japanese adults.^4^^,^^5^ In addition, many lifestyle habits tend to track across childhood and into adulthood.^6^^,^^7^ Thus, identifying modifiable determinants of elevated blood pressure early in life may contribute to preventing hypertension in later adulthood.

Restricted salt and alcohol consumption and increased intake of potassium, fiber, fruits, and vegetables have been reported to reduce blood pressure among adults.^8^^–^^10^ Salt intake has been considered a contributor to elevated blood pressure in children and adolescents; however, the role of other dietary factors in blood pressure remains unclear.^11^^–^^14^ We previously reported that seaweed intake was inversely associated with diastolic blood pressure (DBP) among boys and with systolic blood pressure (SBP) among girls in a cross-sectional study of Japanese children aged 3 to 6 years.^15^ There are some reports that dietary patterns that include seaweed-rich foods are associated with lower blood pressure and decreased arterial stiffness.^16^^,^^17^ Several experimental studies have also shown that feeding on seaweed or its extracts lowers blood pressure in animals.^18^^–^^21^ Alginate extracted from seaweed was reported to attenuate salt-induced hypertension by decreasing cardiovascular and renal damage in rats.^22^^,^^23^ Several kinds of peptides with activity against angiotensin I–converting enzyme (ACE I) have been isolated from seaweed.^24^^,^^25^ However, few intervention studies have investigated the effects of seaweed intake on blood pressure,^26^^–^^29^ and none have been conducted among children.

Here, we conducted an intervention study examining the effect of seaweed intake on blood pressure among preschool-aged Japanese children. Nori (dried laver), an edible seaweed widely consumed in Japan, was chosen as a dietary intervention.

## METHODS

### Study design and participants

This study was an intervention study conducted from April through July 2010. The study protocol and informed consent procedure were approved by the ethics board of Gifu University Graduate School of Medicine, Gifu, Japan. The study was registered at UMIN clinical trials registry on April 21, 2010 (ID: UMIN000003509).

Subjects were second-year students aged 4–5 years attending a preschool in Aichi Prefecture, Japan. Of 99 children, 89 (89.9%) agreed to be enrolled in our study, with their parents providing written informed consent.

### Randomization and blinding

**Figure 1.**
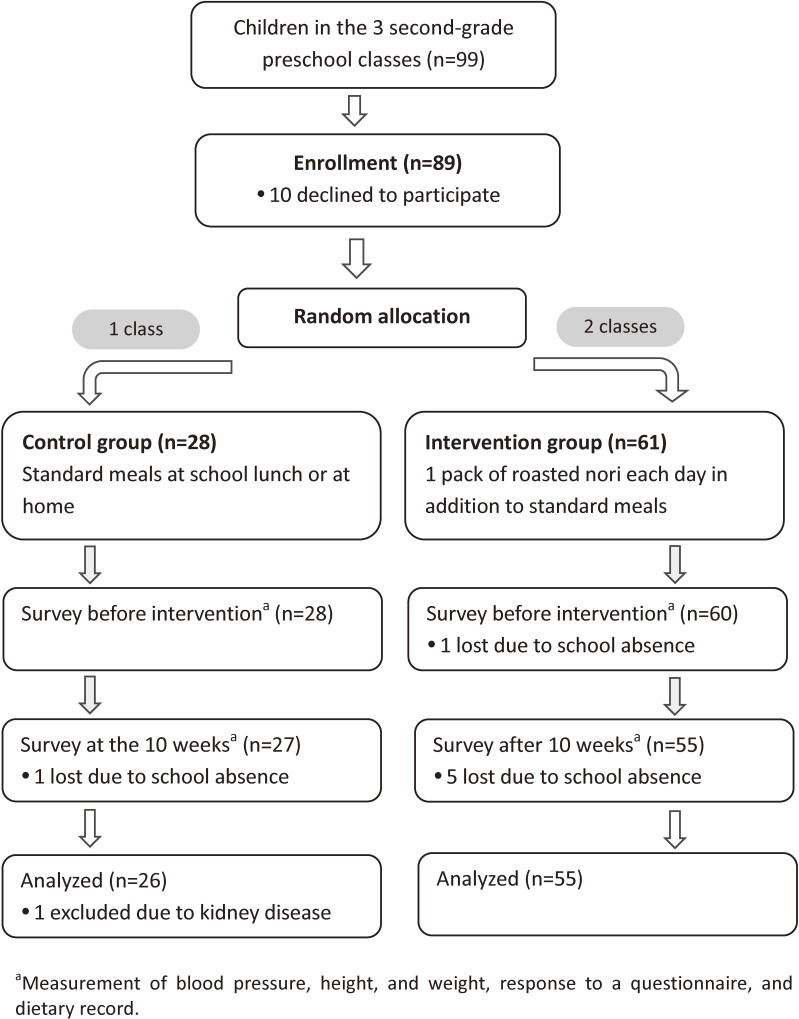
Flow diagram of participants

Figure 1 shows flow diagram of participants. The preschool had three second-grade classes. Among the three classes, two were randomly assigned as the intervention group (61 children), and the other class was assigned as the control group (28 children). The allocation was conducted using a random number table. Although the trial was basically unblinded, the staff who measured blood pressure were not given information on the group assignment.

### Interventions

The intervention period was 10 weeks. In the intervention group, one pack (1.76 grams/6 sheets) of roasted nori (Yamamotoyama, Tokyo, Japan) was served to children in addition to standard meals at school lunch. Roasted nori is a dried seaweed snack that is often consumed alone or with rice in Japan and is generally popular among Japanese children. With the popularization of sushi, nori is becoming more widely consumed in Western countries. The nori given to study participants was made of red seaweed cultivated in the Ariake Sea near the island of Kyushu in southern Japan and did not contain salt or other flavorings. Study staff and preschool teachers encouraged the children to eat all of their lunch foods, including nori, every day. On weekends, children were asked to eat one pack of nori each day at home. In the control group, nothing was added to meals at school lunch or at home.

### Examination

Before the intervention and at the 10th week of the intervention, participant height, weight, and blood pressure were measured. In addition, children’s health status and lifestyle were assessed through a parent-administered questionnaire. Nutritional data, including seaweed intake, were also collected using 3-day dietary records covering 2 consecutive weekdays and 1 weekend day.

### Blood pressure and other measurements

SBP, DBP, and heart rate were measured using an automated sphygmomanometer (ES-H55; Terumo Co., Tokyo, Japan) with oscillometer methods. Measurements were taken midmorning three times successively on the upper arm, using an appropriately sized cuff based on each child’s arm circumference and with subjects in a sitting position after a few minutes of rest. The means of these measurements were used for analysis. Body mass index (BMI) was calculated as (weight in kg)/(height in m)^2^.

### Dietary record and questionnaire

Parents were given written instructions for recording their children’s food intake and recorded the amounts and types of foods and beverages their children consumed during each of the 3 days. If parents had difficulty with recording, our staff assisted them by phone. For school lunches and snacks during the 2 weekdays, dieticians checked meals provided by the preschool and measured the quantity of food each child had left over. Individual nutrient intake was estimated using the Japanese Standard Table of Food Composition, 5th Revised and Enlarged Edition.^30^ According to the nutrient density method, carbohydrate, protein, and fat intake were presented as % of total energy. The other nutrient intakes were presented as grams per 1,000 kcal of total energy.

Child’s birth weight, present illness, and use of medicine were inquired in a parent-administered questionnaire. Information on child’s lifestyles, including sleep, screen viewing, and reading, and smoking habits of family members was also obtained from the questionnaire. Sleep duration at night was calculated as the time from bedtime to wake-up time. Sedentary lifestyle time was defined as the sum of the time children spent watching television, playing video games, or reading. Household smoking was defined as exposure to tobacco smoke due to one or more family members regularly smoking at home.

### Sample size

For power analysis, we used a standard deviation of 11 mm Hg in blood pressure from our previous cross-sectional study of preschool children.^15^ Because there was no similar intervention study among children, we estimated 8 mm Hg decrease in blood pressure based on a previous study of hypertensive Japanese patients in which differences in blood pressure change were 5–10 mm Hg between the intervention and control groups after intake of 5 g/day of seaweed (wakame) powder.^27^ When randomization of 2:1 for the intervention and control group were considered, we calculated that with total sample of 69 participants, the study would have 80% power to detect the difference in blood pressure with a type I error of 5%.

### Statistical analysis

Complete measurement data were obtained for 82 children (Figure 1). After one child with kidney disease was excluded, 55 children in the intervention group and 26 in the control group were analyzed. None of participants had history of or medicine use for hypertension.

Analyses were conducted using SAS 9.4 (SAS Institute, Cary, NC, USA) and were stratified by sex. All *P*-values were calculated using a two-sided test. A significant difference was defined as *P* < 0.05.

Subject characteristics before the intervention were summarized for the intervention and control groups (Table 1). Means and proportions were compared between groups using *t*-test and chi-squared test, respectively. Blood pressure levels and seaweed intake were not different between the intervention and control groups. Boys in the control group were taller than those in the intervention group (*P* = 0.018). Girls in the control group had higher fat intake than did those in the intervention group (*P* = 0.010).

**Table 1.** Characteristics of subjects in the intervention and control groups before the intervention

	Boys				*P*^a^	Girls				*P*^a^
	
	Control		Intervention			Control		Intervention		
*n*	11		28			15		27		
Age, years	4.5	(0.3)	4.5	(0.3)	0.87	4.5	(0.3)	4.5	(0.3)	0.91
Height, cm	105.7	(3.9)	102.1	(4.2)	0.018	101.5	(4.9)	102.5	(4.5)	0.53
Weight, kg	17.5	(2.1)	16.4	(1.6)	0.092	16.6	(2.3)	16.3	(1.8)	0.67
Body mass index, kg/m^2^	15.6	(1.1)	15.7	(0.8)	0.68	16.0	(1.1)	15.5	(0.9)	0.10
Systolic blood pressure, mm Hg	102.7	(9.2)	100.2	(10.9)	0.51	100.3	(11.6)	100.5	(11.4)	0.96
Diastolic blood pressure, mm Hg	65.8	(13.1)	60.6	(9.4)	0.17	60.4	(10.1)	60.0	(8.7)	0.87
Heart rate, beats/min	93.7	(12.4)	88.9	(15.7)	0.37	91.4	(12.4)	94.4	(8.2)	0.37
Birth weight, g	3,310.8	(486.3)	3,028.2	(544.7)	0.14	2,925.5	(711.8)	3,139.5	(483.2)	0.25
Present illness, %	18.2%		14.8%		0.80	20.0%		14.8%		0.67
Medicine use, %	30.0%		28.6%		0.93	14.3%		26.9%		0.36
Household smoking, %	45.5%		71.4%		0.13	60.0%		70.4%		0.49
Sleep time at night, h	10.1	(0.5)	10.0	(0.6)	0.52	10.0	(0.5)	10.2	(0.5)	0.44
Sedentary lifestyle time, minutes	100.5	(42.1)	122.5	(61.4)	0.28	127.3	(75.4)	100.9	(42.0)	0.15
Dietary intake										
Total energy, kcal/d	1,391.3	(184.5)	1,349.5	(229.2)	0.59	1,365.2	(198.5)	1,314.7	(160.1)	0.37
Carbohydrate, % energy	55.55	(4.9)	56.77	(4.9)	0.49	54.63	(4.2)	57.30	(4.4)	0.063
Protein, % energy	13.36	(1.6)	12.28	(2.1)	0.13	12.24	(1.3)	12.99	(1.1)	0.052
Fat, % energy	30.03	(4.1)	29.80	(3.9)	0.87	32.26	(4.0)	28.83	(3.8)	0.010
Salt, g/1000 kcal/d	3.53	(0.4)	3.28	(0.6)	0.20	3.41	(0.7)	3.63	(0.6)	0.32
Seaweed, g/1000 kcal/d	1.4	(1.1)	1.1	(1.2)	0.43	1.0	(0.9)	1.2	(0.7)	0.41
Seaweed, once/day or more, %	27.3%		32.1%		0.77	20.0%		18.5%		0.91
Roasted laver, once/day or more, %	18.2%		14.3%		0.76	6.7%		3.7%		0.67

mean (standard deviation) or percentage.

^a^*P* values by *t*-test or *chi*-squared test.

To assess the effect of nori intake on blood pressure levels, we compared changes in SBP and DBP between the intervention and control groups by one-way analysis of covariance. Because blood pressure levels before the intervention would likely be associated with the changes afterward, SBP and DBP before the study were controlled for (model 1). As a sensitivity analysis, additional adjustment for height and fat intake was conducted (model 2), because the differences in these variables observed between the intervention and control groups.

## RESULTS

In boys, SBP decreased by 8.29 mm Hg from before the intervention to the 10th week of the intervention in the intervention group, while an increase of 0.50 mm Hg was observed in the control group (*P* for difference in change = 0.051) (model 1) (Table 2). The changes in DBP level were −6.77 mm Hg in the intervention group and −0.05 mm Hg in the control group (*P* = 0.031). In girls, no difference in blood pressure changes was found between the intervention and control groups (*P* for SBP = 0.85 and *P* for DBP = 0.27). In sensitivity analyses adjusting additionally for height and fat intake, the difference in DBP changes remained significant between in the intervention group and in the control group among boys (*P* = 0.014).

**Table 2.** Blood pressure levels among the intervention and control groups

	Blood pressure			Change in blood pressure					
	
	*n*	before	after 10 weeks	crude		model 1		model 2	
		
				mean	SE	mean	SE	mean	SE
Boys									
SBP									
control	11	102.7	102.0	−0.76	4.23	0.50	3.67	−0.46	4.01
intervention	28	100.2	92.4	−7.80	2.65	−8.29	2.30	−7.92	2.41
*P*^a^				0.17		0.051		0.13	
DBP									
control	11	65.8	63.6	−2.21	3.05	−0.05	2.51	1.25	2.70
intervention	28	60.6	54.7	−5.92	1.91	−6.77	1.56	−7.28	1.60
*P*^a^				0.31		0.031		0.014	

Girls									
SBP									
control	15	100.3	97.3	−3.07	2.91	−3.13	2.39	−2.46	2.59
intervention	27	100.5	97.9	−2.60	2.17	−2.57	1.78	−2.94	1.88
*P*^a^				0.90		0.85		0.89	
DBP									
control	15	60.4	56.2	−4.29	2.65	−4.05	1.96	−3.91	2.14
intervention	27	60.0	58.8	−1.16	1.98	−1.29	1.46	−1.37	1.55
*P*^a^				0.35		0.27		0.37	

DBP, diastolic blood pressure; SBP, systolic blood pressure; SE, standard error.

^a^*P* value by analysis of variance or analysis of covariance.

model 1: adjustment for blood pressure level (SBP or DBP) before the intervention.

model 2: adjustment for blood pressure level (SBP or DBP) before the intervention, height, and fat intake.

Except for nori supplied by study staff, seaweed intake at the 10th week of the intervention was not different between the intervention and control groups in either boys or girls. For boys in the intervention group, total energy intake decreased by 111.3 kcal/d from before the intervention to the 10th week, while such a change was not observed in the control group (*P* for difference in change = 0.063). After additional adjustment for the change in total energy intake from before the intervention to the 10th week of the intervention, changes in DBP were −7.37 mm Hg in the intervention group and 1.48 mm Hg in the control group (*P* = 0.017).

Three children reported the use of medicine with a sympathomimetic effect, such as tulobuterol hydrochloride and DL-methylephedrine hydrochloride, for treatment of a cold during the study period. Results were not substantially altered when these subjects were excluded from the analysis. For example, the changes in DBP from before the intervention to the 10th week were −6.70 mm Hg in the intervention group and 0.11 mm Hg in the control group (*P* = 0.033) (model 1).

## DISCUSSION

To our knowledge, this is the first intervention study to suggest that seaweed consumption has beneficial effects on blood pressure among healthy children. We observed that addition of 1.76 grams per day of nori to the diet over 10 weeks significantly decreased DBP in boys. Four previous intervention studies on seaweed intake and blood pressure have been conducted among adults. A decrease in mean blood pressure was observed among Swedish patients with mild hypertension given 12 and 24 g/day of seaweed fiber for 4 weeks compared with placebo treatment.^26^ In hypertensive Japanese patients, SBP and DBP decreased compared with the control group after intake of 5 g/day of seaweed (wakame) powder for 8 weeks.^27^ Among patients with high blood pressure in Ecuador, a decrease in SBP from baseline was observed after intake of 4 g/day of dried seaweed (wakame) for 4 weeks and followed by 6 g/day for 4 weeks, although comparison with a placebo group was not shown.^28^ Another uncontrolled intervention study reported that nori-derived peptide intake of 1.8 g/day induced a significant blood pressure reduction in hypertensive but not normotensive subjects.^29^ Although these results suggest that seaweed consumption may help prevent increased blood pressure, further studies are warranted to confirm the anti-hypertensive effects of seaweed intake.

Peptides derived from *Pyropia yezoensis* have been shown to have ACE I inhibitory activity and hypotensive effects.^29^^,^^31^ Porphyran, a main constituent of *Pyropia yezoensis*, potentially has antioxidant activity.^25^ It is likely that these unique biologically active compounds of nori play an important role on reducing blood pressure, although we could not identify a causative factor in this dietary intervention. Nori also has high contents of dietary fiber, protein, minerals, vitamins, and polyunsaturated fatty acids.^30^^,^^32^ Protein content in red and green seaweed is generally higher than that in brown seaweed, which is much greater than that found in high-protein legumes, such as soybeans.^32^ The mineral content of seaweed is higher than that of most land vegetables.^32^ It is possible that calcium and magnesium, as well as potassium, are responsible for lowering blood pressure.^33^ Nori is also known to contain abundant vitamin B12 and folic acid, whereas other edible seaweeds contain little or none of these vitamins. Folic acid may reduce homocysteine level and improve endothelial function, resulting in reduced blood pressure.^34^^,^^35^ Nori is particularly rich in eicosapentaenoic acids, which have beneficial effects on cellular and molecular mechanisms of atherosclerosis.^36^ The combined intake of these components in seaweed might exert positive effects on blood pressure.

Although our previous cross-sectional study showed an inverse association between seaweed intake and SBP in girls, a blood pressure-lowering effect of seaweed intake was not observed in girls in this intervention study. Considering that a longer-term diet was evaluated in the cross-sectional study, it is possible that more time is required for seaweed intake to influence blood pressure in girls; however, longitudinal studies on daily seaweed intake would be required to confirm this. Although our findings also suggest a sex difference in the effects of seaweed intake on blood pressure, the mechanisms are unknown. As the release of vasopressin and aldosterone induced by angiotensin II and the fluid intake response to angiotensin II were reported to differ by sex,^37^ the effect of nori-derived peptides on the renin-angiotensin-aldosterone system may also be influenced by sex.

The merits of our study include the interventional design, high participation rate, repeated measurement of blood pressure, and use of dietary records. Because the nori intake intervention was done at school lunch in the presence of teachers and study staff, most children consumed the nori as directed. However, we were not able to guarantee the completeness of nori intake on weekends. In addition, the nori trial was unblinded for study subjects. Because we randomly allocated the intervention or control groups to the units of school class, but not to individual children, differences in some characteristics (height and fat intake) were observed between the intervention and control groups. However, the results were not substantially altered after additional adjustment for height and fat intake. We could not verify the effect of nori using the other designs, such as a cross-over study. Finally, we need to be cautious of comparing the values of blood pressure we observed with those of previous studies, because the methods, devices, and environment of measurement may affect the values.^38^ In addition, the data of blood pressure measurements is still scarce among preschool Japanese children.^15^^,^^35^^,^^39^^–^^41^

In conclusion, this intervention study showed that nori intake lowered DBP in boys aged 4 to 5 years. Although our findings suggest that seaweed intake potentially decreases blood pressure in childhood, future prospective research is needed to determine the preventive effects of seaweed intake early in life on hypertension in later adulthood.
